# Dengue Vaccines in a Changing Epidemiological Landscape: Current Evidence, Unresolved Challenges, and Public Health Considerations

**DOI:** 10.3390/vaccines14090729

**Published:** 2026-08-24

**Authors:** Susanna Esposito, Nicola Principi

**Affiliations:** 1 Pediatric Clinic, Department of Medicine and Surgery, University Hospital of Parma, 43126 Parma, Italy; 2 Department of Pathophysiology and Transplantation, Università Degli Studi di Milano, 20122 Milan, Italy; nicola.principi@unimi.it

**Keywords:** dengue, dengue vaccines, antibody-dependent enhancement, tetravalent immunity, vaccine efficacy, public health implementation

## Abstract

Background: Dengue has expanded rapidly beyond traditional tropical and subtropical regions, driven by climate change, urbanization, population mobility, and the spread of competent Aedes vectors. Vaccination is an increasingly important component of dengue prevention, but development has been complicated by four viral serotypes, antibody-dependent enhancement, variable baseline serostatus, and the need for balanced and durable tetravalent immunity. Methods: We conducted a narrative review of PubMed/MEDLINE, Google Scholar, ClinicalTrials.gov, and relevant public health and regulatory sources. Evidence on dengue epidemiology, immunopathogenesis, licensed vaccines, advanced candidates, efficacy, immunogenicity, safety, durability, and implementation was critically evaluated. Priority was given to randomized trials, long-term follow-up studies, regulatory assessments, and surveillance data. Evidence was synthesized descriptively without formal meta-analysis or risk-of-bias assessment. Results: CYD-TDV was the first licensed dengue vaccine but is restricted to individuals with documented previous infection because seronegative recipients may experience an increased risk of severe dengue. TAK-003 has demonstrated overall efficacy against virologically confirmed dengue and dengue-related hospitalization in both baseline-seropositive and baseline-seronegative populations. However, protection is heterogeneous by serotype, and evidence remains limited or uncertain for some serotype-by-serostatus strata. Butantan-DV offers a promising single-dose strategy, but broader use requires additional long-term safety, effectiveness, and serotype-specific data. Inactivated, DNA, viral-vectored, virus-like particle, and mRNA vaccines remain investigational. Conclusions: Dengue vaccination should be integrated with surveillance, vector control, clinical preparedness, and risk communication. Population-based vaccination is most appropriate in high-transmission settings, whereas selective, risk-based strategies are preferable in temperate regions. Continued pharmacovigilance and effectiveness monitoring are essential to guide safe and equitable implementation.

## 1. Introduction

Dengue is a mosquito-borne viral disease caused by four antigenically distinct dengue virus serotypes and transmitted primarily by *Aedes aegypti* and, increasingly, *Aedes albopictus*. Historically, dengue transmission was concentrated in tropical and subtropical regions, where climatic and ecological conditions supported vector survival and sustained viral circulation. Over the past five decades, however, dengue has undergone an unprecedented geographic expansion, evolving from a disease endemic in fewer than ten countries into a major global public health threat affecting more than 130 countries worldwide [1,2,3].

This expansion reflects the complex interplay of environmental, demographic, and socioeconomic determinants. Climate change is increasing the geographic and seasonal suitability of many regions for *Aedes* mosquitoes by extending periods favorable for vector reproduction, survival, and viral transmission. At the same time, rapid and often unplanned urbanization, population growth, globalization, inadequate vector-control infrastructure, and increasing international travel have facilitated mosquito proliferation and the dissemination of dengue viruses across national and continental boundaries [4,5].

Consequently, more than half of the world’s population is now estimated to live in areas at risk of dengue infection [6]. The associated disease burden has risen dramatically, with substantial clinical, social, and economic consequences for affected populations and healthcare systems. According to the World Health Organization (WHO), 2024 marked the highest number of dengue cases ever reported globally, with approximately 14.4 million notified cases, including more than 7.7 million laboratory-confirmed infections, 52,738 severe cases, and over 11,000 deaths [6]. Although the greatest burden remains concentrated in Latin America, Southeast Asia, and the Western Pacific region, increasing international mobility and the expansion of competent mosquito vectors are progressively exposing previously non-endemic areas to both imported infections and autochthonous transmission [6].

This epidemiological transition is particularly evident in temperate regions. In the United States, the Centers for Disease Control and Prevention reported a record 3798 dengue cases in 2024, representing a 359% increase compared with the average annual incidence recorded between 2010 and 2023. Although most infections were travel-associated, locally acquired cases continued to occur in Florida, Texas, and California, where competent vectors are established [7]. Europe has experienced a similar shift. Once considered almost exclusively a destination for imported dengue cases, the region has documented an increasing number of autochthonous transmission events. France, Spain, Portugal, Croatia, and Italy have all reported locally acquired infections, largely in association with the widespread establishment of *Aedes albopictus* and increasingly favorable climatic conditions for arboviral transmission [8,9,10,11].

These events illustrate the growing vulnerability of Mediterranean countries and demonstrate that dengue can no longer be regarded solely as an imported tropical disease. They also underscore the need for integrated prevention strategies combining epidemiological and entomological surveillance, timely case detection, effective vector control, public health preparedness, travel-medicine interventions, risk communication, and vaccination where clinically and epidemiologically appropriate.

Within this rapidly evolving epidemiological context, the development of safe and effective vaccines has become an increasingly important public health priority. However, dengue vaccine development has proved substantially more complex than initially anticipated because of the biological characteristics of the virus and the distinctive nature of the host immune response. The coexistence of four serotypes, the requirement for balanced and durable tetravalent protection, the potential for antibody-dependent enhancement, differences in baseline serostatus, and uncertainties regarding correlates of protection have complicated both vaccine design and clinical evaluation. Consequently, only a limited number of vaccine candidates have progressed to licensure, and important questions remain regarding long-term effectiveness, serotype-specific protection, safety in dengue-naïve individuals, optimal target populations, and the most appropriate strategies for vaccine implementation.

This narrative review summarizes the principal virological and immunological challenges that have shaped dengue vaccine development. It critically evaluates licensed and authorized vaccines, as well as candidates in advanced clinical development, with particular attention to efficacy, immunogenicity, safety, durability of protection, and performance according to serotype and baseline serostatus. Finally, it examines the scientific, clinical, and public health issues that must be addressed to support evidence-based vaccine recommendations and to advance toward the goal of safe, effective, durable, and broadly applicable dengue vaccination.

## 2. Methods

A comprehensive literature search was conducted in PubMed/MEDLINE, Google Scholar, and ClinicalTrials.gov. Relevant documents were also retrieved from the websites of international and national public health and regulatory authorities, including the World Health Organization (WHO), the Centers for Disease Control and Prevention (CDC), the European Centre for Disease Prevention and Control, the European Medicines Agency, the US Food and Drug Administration, and national regulatory and surveillance agencies. The search covered the period from 1 January 2000 to 31 March 2026; relevant regulatory and public health updates released during manuscript revision were additionally reviewed through July 2026.

Search terms were used individually and in combination and included “dengue,” “dengue fever,” “dengue virus,” “DENV,” “dengue virus serotypes,” “DENV-1,” “DENV-2,” “DENV-3,” “DENV-4,” “dengue viral proteins,” “dengue immunology,” “antibody-dependent enhancement,” “neutralizing antibodies,” “cell-mediated immunity,” “dengue vaccine,” “dengue vaccination,” “vaccine efficacy,” “vaccine effectiveness,” “vaccine safety,” and the names of individual vaccine candidates and platforms. Additional publications were identified through manual screening of the reference lists of relevant original studies, reviews, consensus statements, and public health guidance documents.

Priority was given to peer-reviewed original research, including randomized controlled trials, long-term follow-up studies, observational effectiveness studies, immunogenicity and safety studies, human challenge studies, and investigations addressing dengue virology and immunopathogenesis. Phase III efficacy trials and their extended follow-up analyses were considered particularly relevant when assessing licensed or authorized vaccines. Preclinical and early-phase clinical studies were included for vaccine platforms that had not yet reached pivotal efficacy evaluation. Systematic and narrative reviews, surveillance reports, regulatory assessments, position papers, and public health recommendations were used to provide epidemiological, clinical, and policy context and to address areas for which primary evidence was limited or still evolving.

The literature was evaluated with particular emphasis on study design, population characteristics, baseline dengue serostatus, age, geographic setting, circulating serotypes, duration of follow-up, clinical endpoints, and the precision and consistency of efficacy and safety estimates. When evidence was incomplete or conflicting, greater weight was assigned to larger controlled studies, longer follow-up periods, peer-reviewed data, and assessments issued by independent public health or regulatory authorities.

Because this was a narrative review rather than a systematic review, study identification and selection were not conducted according to a prespecified systematic-review protocol, and no formal risk-of-bias assessment, quantitative evidence grading, or meta-analysis was performed. Evidence was synthesized descriptively and interpreted according to its biological plausibility, methodological robustness, clinical relevance, and implications for vaccination policy and practice. Particular emphasis was placed on areas of uncertainty, including protection in dengue-seronegative individuals, serotype-specific and genotype-specific effectiveness, durability of immunity, the potential risk of vaccine-associated disease enhancement, and the applicability of vaccination strategies across endemic and non-endemic settings.

## 3. Immunological and Virological Challenges in Dengue Vaccine Development

### 3.1. Antigenic Diversity and the Need for Balanced Tetravalent Immunity

The development of a safe and effective dengue vaccine has proved considerably more complex than vaccine development for most other viral infections because of the distinctive virological and immunological features of dengue virus (DENV). DENV belongs to the genus *Flavivirus* and comprises four antigenically distinct but genetically related serotypes: DENV-1, DENV-2, DENV-3, and DENV-4. Infection with one serotype generally induces long-lasting, and probably lifelong, homologous immunity, but provides only transient and incomplete protection against the remaining serotypes. Individuals living in endemic areas may therefore experience multiple dengue infections during their lifetime [12,13,14].

A central objective of dengue vaccine development is consequently the induction of balanced, durable, and protective immunity against all four serotypes. In contrast to vaccines directed against viruses with limited antigenic diversity, dengue vaccines cannot depend on strong protection against only one or two serotypes. They must generate sufficiently potent and persistent immune responses to each serotype while avoiding marked immunodominance of individual vaccine components. Inadequate or unbalanced tetravalent immunity may result in heterogeneous serotype-specific protection and leave vaccine recipients susceptible to infection with poorly covered serotypes. This requirement has represented one of the principal obstacles throughout dengue vaccine development and helps explain the variable performance observed across vaccine platforms, populations, and epidemiological settings [14,15,16].

### 3.2. Antibody-Dependent Enhancement and Safety Concerns

The risk of antibody-dependent enhancement (ADE) constitutes a further and uniquely important challenge. During infection with a heterologous serotype, pre-existing cross-reactive antibodies that are non-neutralizing or present at sub-neutralizing concentrations may bind to the virus without preventing infection. The resulting immune complexes can facilitate viral entry into Fcγ receptor-bearing cells, including monocytes, macrophages, and dendritic cells, potentially increasing viral replication and amplifying inflammatory and immunopathological responses. This mechanism has been associated with an increased risk of severe dengue, including dengue hemorrhagic fever and dengue shock syndrome [17]. Epidemiological and immunological studies have further shown that intermediate concentrations of cross-reactive antibodies may be associated with the greatest risk of severe disease, supporting the biological relevance of ADE in humans [16]. Importantly, emerging evidence suggests that dengue virus-specific IgA responses may mitigate ADE. Unlike IgG, IgA inefficiently engages Fcγ receptors and does not appear to mediate enhancement of infection. Instead, DENV-specific IgA can neutralize viral particles and competitively inhibit the binding of enhancement-prone IgG antibodies, thereby reducing the formation of pathogenic immune complexes. Experimental studies have demonstrated that monomeric IgA antagonizes IgG-mediated ADE and attenuates pro-inflammatory cytokine responses in Fc receptor-bearing cells, supporting a protective, non-pathological role for IgA during dengue infection [18,19]. Dengue vaccines must therefore do more than induce measurable antibody responses: they should generate durable, functionally neutralizing antibodies against all four serotypes while minimizing immune profiles that could theoretically enhance subsequent infection.

### 3.3. Complexity of Protective Antibody Responses and Absence of Correlates of Protection

The complexity of antibody-mediated protection is compounded by the structural characteristics of the dengue envelope (E) glycoprotein. The E protein is the principal target of neutralizing antibodies, but its antigenic surface contains linear, conformational, and quaternary epitopes that differ markedly in accessibility, immunodominance, and protective capacity. Studies using human monoclonal antibodies have demonstrated that some of the most potent neutralizing responses recognize complex quaternary epitopes displayed only on mature, intact virions rather than simple linear sequences or isolated recombinant proteins [20,21]. Moreover, the structural heterogeneity of dengue virions, including differences in maturation state and temperature-dependent conformational dynamics, can alter epitope exposure and antibody binding. These observations suggest that vaccine platforms capable of reproducing or preserving native virion architecture may be more likely to induce effective neutralizing responses than approaches based exclusively on isolated antigens.

Neutralizing antibody titers are widely used as markers of vaccine immunogenicity, but they do not constitute a fully validated correlate of protection. Assay results may vary according to the viral strain, cell substrate, complement conditions, and testing methodology, and high antibody titers do not invariably translate into equivalent clinical protection across serotypes or populations. Conversely, protection may also depend on antibody quality, epitope specificity, Fc-mediated functions, and the contribution of cellular immunity. The absence of a universally accepted immunological correlate complicates vaccine candidate selection, comparison across clinical trials, and the interpretation of long-term protection.

### 3.4. The Role of Cellular Immunity

Beyond humoral immunity, cell-mediated responses are increasingly recognized as important determinants of protection and disease modulation. Both CD4+ and CD8+ T cells contribute to viral clearance, immune regulation, and the development of immunological memory. Relevant T-cell epitopes are located not only in structural proteins but also in highly conserved non-structural proteins, particularly NS1, NS3, and NS5 [22,23]. Multifunctional T-cell responses have been associated with favorable clinical outcomes and may contribute to cross-serotype immunity. Vaccine platforms that induce broad dengue-specific cellular responses in addition to neutralizing antibodies may therefore offer advantages in terms of durability and heterotypic protection [22,23]. This consideration is particularly relevant when comparing vaccines that contain dengue non-structural proteins with chimeric platforms in which these proteins derive from another flavivirus backbone.

### 3.5. Host-Related Factors and Variability in Vaccine Response

Baseline immune status represents another major determinant of vaccine performance. Prior dengue infection may enhance vaccine-induced responses through immunological priming, whereas vaccination of dengue-naïve individuals may produce qualitatively different antibody and T-cell profiles. Experience with licensed vaccines has demonstrated that efficacy and safety can vary substantially according to baseline serostatus. Age, previous exposure to other flaviviruses, maternal antibodies in young children, and the intensity of dengue transmission may further modify immune responses and clinical outcomes. These factors complicate the identification of universally appropriate target populations and may necessitate different vaccination strategies across endemic and non-endemic settings.

### 3.6. Viral Genetic Diversity and Evolution

Viral genetic diversity adds an additional layer of complexity. Each DENV serotype includes multiple genotypes and lineages that differ geographically and continue to evolve. Although immunity is generally expected to provide protection within a serotype, genetic variation may influence viral fitness, antigenicity, replication capacity, and susceptibility to vaccine-induced antibodies. Such differences may contribute to geographic variation in vaccine effectiveness and to changes in performance over time as circulating strains evolve [2,24]. Continuous molecular and epidemiological surveillance is therefore essential during vaccine evaluation and implementation, particularly in settings where serotype replacement or genotype shifts occur.

### 3.7. Technical Challenges in Vaccine Design and Formulation

The interactions among the four components of tetravalent live vaccines also present important technical challenges. Differences in replication fitness among vaccine strains may lead to viral interference, immunological dominance, and unequal immune responses. Achieving appropriate attenuation without compromising immunogenicity requires careful balancing of the individual components. Excessive attenuation may reduce immune stimulation, whereas insufficient attenuation may increase reactogenicity or safety concerns. These challenges are less pronounced in non-replicating platforms but may be replaced by lower immunogenicity, the need for adjuvants, or repeated dosing. Collectively, these factors demonstrate that dengue vaccine development requires simultaneous consideration of viral diversity, host immune status, antibody quality, cellular immunity, and programmatic feasibility. The principal challenges and their implications for vaccine design are summarized in Table 1.

An ideal dengue vaccine should therefore provide durable protection against all four serotypes and clinically relevant genotypes, be effective and safe in both seropositive and seronegative individuals, minimize any risk of vaccine-associated disease enhancement, and induce broad humoral and cellular immunity. It should also protect against severe disease and hospitalization, retain effectiveness across age groups and epidemiological settings, and be compatible with a simple, affordable, and programmatically feasible vaccination schedule.

Current vaccine strategies—including live-attenuated, purified inactivated, recombinant subunit, viral-vectored, virus-like particle, DNA, and mRNA-based platforms—seek to address these interconnected challenges through different immunological and technological approaches. However, the complexity of dengue immunopathogenesis, the absence of a definitive correlate of protection, and the need to achieve balanced tetravalent immunity largely explain why vaccine development has required decades of research and why only a limited number of candidates have progressed to licensure or advanced clinical evaluation.

## 4. Licensed or Authorized Dengue Vaccines

Three dengue vaccines have reached regulatory authorization, although they differ substantially in platform, schedule, target population, requirement for pre-vaccination screening, and supporting evidence. Their principal characteristics, advantages, limitations, and current regulatory positioning are compared in Table 2 [25,26,27,28,29,30,31,32,33,34,35,36,37,38,39,40,41,42,43,44,45,46,47,48,49,50,51,52,53,54,55].

### 4.1. CYD-TDV (Dengvaxia^®^, Sanofi Pasteur)

CYD-TDV (Dengvaxia^®^, Sanofi Pasteur, Lyon, France) was the first dengue vaccine to enter clinical use. Developed during the 1990s, it is a live-attenuated, chimeric tetravalent vaccine constructed using the licensed yellow fever 17D vaccine virus as a genetic backbone. Through recombinant DNA technology, the genes encoding the premembrane (prM) and envelope (E) proteins of each of the four dengue virus serotypes were inserted into the yellow fever 17D backbone, generating four chimeric vaccine viruses that were subsequently combined into a tetravalent formulation.

This strategy was designed to exploit the well-established safety profile, genetic stability, and manufacturing experience associated with the yellow fever 17D vaccine while inducing neutralizing antibodies against all four DENV serotypes. However, because CYD-TDV contains dengue prM and E proteins but not yellow fever-derived non-structural proteins, the immune response is directed predominantly toward dengue structural antigens. Consequently, the vaccine induces relatively limited dengue-specific T-cell responses compared with natural infection or vaccine platforms that include dengue non-structural proteins.

Preclinical studies demonstrated that CYD-TDV was genetically stable, immunogenic, capable of inducing neutralizing antibody responses against all four serotypes, and not associated with significant neurovirulence or major safety concerns in animal models [25,26]. These findings provided the basis for advancing the vaccine into early-phase clinical evaluation.

Initial phase I studies in healthy flavivirus-naïve adults showed an acceptable safety profile. Most adverse events were mild and included injection-site reactions, headache, myalgia, and transient fever, with no vaccine-related serious adverse events reported. These studies also confirmed that CYD-TDV could induce neutralizing antibodies against all four DENV serotypes. However, the magnitude of the response was not balanced across serotypes, with generally stronger responses against DENV-3 and DENV-4 and weaker responses against DENV-2. This pattern persisted throughout subsequent clinical development [26].

Phase II studies were conducted in endemic and non-endemic settings across Asia and Latin America and included children, adolescents, and adults. Several vaccination schedules were evaluated, and a three-dose regimen administered at 0, 6, and 12 months was selected because it produced the most consistent immune responses. Neutralizing antibody titers increased after successive doses and remained detectable for several years. An integrated analysis of ten phase II and six phase III studies demonstrated persistence of neutralizing antibodies for up to four years after completion of the vaccination schedule [27].

These studies also revealed marked heterogeneity in immune responses according to both serotype and baseline dengue serostatus. Participants with evidence of previous dengue infection consistently developed higher neutralizing antibody titers than seronegative individuals. This finding was initially regarded as favorable for vaccine use in endemic populations, although its implications for long-term efficacy and safety were not fully recognized at the time.

Regulatory approval was based primarily on two pivotal phase III trials. The CYD14 trial, conducted in five Asian countries, enrolled 10,275 children aged 2–14 years and reported an overall vaccine efficacy of 56.5% against virologically confirmed symptomatic dengue [28]. The CYD15 trial enrolled 20,869 children and adolescents aged 9–16 years in Latin America and demonstrated an overall efficacy of 60.8% [29]. A pooled analysis of both trials showed an overall efficacy of 60.3%, increasing to 65.6% among participants aged ≥9 years, together with significant reductions in dengue-related hospitalization and severe dengue [30].

Vaccine efficacy was not uniform across serotypes. Protection was highest against DENV-3 and DENV-4, intermediate against DENV-1, and lowest against DENV-2. Efficacy was also consistently greater in participants who were dengue-seropositive at baseline than in those who were seronegative.

Long-term follow-up substantially altered the assessment of the vaccine’s benefit–risk profile. Extended surveillance identified an increased risk of hospitalized dengue among younger vaccine recipients, particularly children aged 2–5 years enrolled in the Asian trial. Subsequent retrospective analyses, using a serological assay capable of estimating baseline dengue serostatus, demonstrated that both efficacy and safety were strongly influenced by previous dengue exposure.

Participants with evidence of prior dengue infection experienced substantial and durable protection against symptomatic dengue, hospitalization, and severe disease. In contrast, baseline-seronegative recipients had an increased long-term risk of hospitalization and severe dengue following subsequent natural infection [31,32]. It was shown that among seronegative participants aged ≥9 years, cumulative incidences of hospitalized and severe dengue were 21.1 and 4.5 per 1000 vaccine recipients, respectively, compared with 16.4 and 2.0 per 1000 in placebo recipients. The excess risk was even greater among seronegative children <9 years of age, reaching 79.8 hospitalizations and 17.2 severe dengue cases per 1000 vaccinees, compared with 44.1 and 6.8 per 1000 in controls [33]. These observations led to the “silent primary infection” hypothesis. According to this model, vaccination of a dengue-naïve individual may immunologically resemble an inapparent primary dengue infection. A subsequent natural infection may then behave immunologically as a secondary heterologous infection, with a potentially increased risk of ADE and severe disease.

CYD-TDV received its first marketing authorizations in 2015 and was subsequently approved in several dengue-endemic countries in Asia and Latin America. Following recognition of the serostatus-dependent safety signal, however, the World Health Organization revised its recommendations. Current guidance supports vaccination only in individuals with laboratory-confirmed previous dengue infection, preferably within a pre-vaccination screening or “screen-and-vaccinate” strategy [34].

Regulatory recommendations in the United States and Europe adopted a similarly restricted approach. In 2019, the US Food and Drug Administration approved Dengvaxia for children and adolescents aged 9–16 years with laboratory-confirmed previous dengue infection who lived in endemic US territories, including Puerto Rico [35]. In the European Union, the vaccine was authorized for individuals aged 6–45 years with test-confirmed previous dengue infection. In October 2025, however, the European marketing authorization was withdrawn at the request of the manufacturer for commercial reasons rather than because of newly identified safety concerns [36].

Several limitations continue to restrict the public health use of CYD-TDV. These include the need for reliable pre-vaccination serological screening, the risk of harm in dengue-seronegative recipients, reduced efficacy against DENV-2, relatively limited induction of dengue-specific T-cell responses, and the requirement for a three-dose schedule administered over 12 months [12,13]. The practical performance of a screen-and-vaccinate strategy is also constrained by the imperfect sensitivity and specificity of available serological assays, particularly in populations exposed to other flaviviruses. Laboratory-based tests such as the EUROIMMUN Anti-Dengue Virus NS1 IgG ELISA and Panbio Dengue IgG ELISA demonstrate high specificity (98.8% and 99.2%, respectively), while rapid diagnostic tests (RDTs) show more variable performance, with specificities ranging from 96.0% for SD Bioline to 99.5% for the CTK OnSite Dengue IgG RDT. Cross-reactivity with antibodies induced by Zika virus infection or yellow fever and Japanese encephalitis vaccination may reduce specificity and increase false-positive classifications. No currently available assay achieves the ideal combination of very high sensitivity and specificity; consequently, false-negative results may exclude previously infected individuals who could benefit from vaccination, whereas false-positive results may expose dengue-naïve individuals to unnecessary risk. Additional limitations include the absence of a universally accepted serological gold standard and variability in test performance according to local dengue seroprevalence [37,38]. Moreover, it should be considered that pre-vaccination screening is a major determinant of the cost-effectiveness of CYD-TDV because it introduces additional expenditures for diagnostic testing, laboratory infrastructure, and program implementation, while simultaneously influencing vaccine effectiveness through test accuracy. High screening costs increase overall program costs, whereas imperfect sensitivity and specificity reduce health benefits through false-negative and false-positive classifications. Consequently, the economic value of CYD-TDV is strongly influenced by assay performance, screening costs, and local seroprevalence, with the most favorable cost-effectiveness observed in high-transmission settings [39].

Despite these unsolved problems, CYD-TDV remains a landmark in dengue vaccinology. Its development provided the first proof that vaccination could reduce symptomatic and severe dengue in previously exposed populations, while its long-term safety findings demonstrated the critical importance of baseline serostatus, immune quality, and post-licensure surveillance. These lessons have had a major influence on the design, evaluation, and implementation of subsequent dengue vaccines.

### 4.2. TAK-003 (Qdenga^®^, Takeda)

TAK-003 (Qdenga^®^, Takeda, Tokyo, Japan), the second dengue vaccine to receive regulatory authorization, was developed to address several limitations associated with CYD-TDV, particularly the need for protection in dengue-seronegative individuals and the practical constraints imposed by pre-vaccination serological screening. TAK-003 is a live-attenuated tetravalent vaccine composed of an attenuated DENV-2 strain and three chimeric viruses in which the premembrane and envelope genes of DENV-1, DENV-3, and DENV-4 are expressed on the attenuated DENV-2 genetic backbone [40]. This design preserves the non-structural proteins of DENV-2 in all four vaccine components and is intended to induce both neutralizing antibody responses and dengue-specific cellular immunity.

Preclinical studies demonstrated appropriate attenuation, genetic stability, induction of neutralizing antibodies against all four serotypes, and protection against viral challenge in non-human primates, thereby supporting progression to clinical evaluation [40]. These studies also provided preliminary reassurance regarding safety and did not identify evidence suggestive of vaccine-associated disease enhancement.

The clinical development program included several phase I and phase II trials conducted in both endemic and non-endemic settings [39,40,41,42,43,44,45,46,47]. These studies showed that a two-dose regimen administered three months apart was generally well tolerated and induced neutralizing antibodies against all four DENV serotypes, although the magnitude and persistence of the responses varied by serotype. Importantly, immunogenicity was demonstrated in both dengue-seropositive and dengue-seronegative participants, an essential objective in view of the safety concerns that had emerged with CYD-TDV.

The efficacy of TAK-003 was evaluated in the pivotal TIDES trial, a multicenter, randomized, double-blind, placebo-controlled phase III study involving 20,099 healthy children and adolescents aged 4–16 years from eight dengue-endemic countries in Asia and Latin America. Participants received two vaccine doses three months apart and were prospectively followed for virologically confirmed dengue, dengue-related hospitalization, severe dengue, and adverse events. The inclusion of both seropositive and seronegative participants at baseline was a major strength of the trial and allowed vaccine performance to be assessed according to previous dengue exposure.

During the first 12 months after completion of the two-dose schedule, TAK-003 demonstrated an efficacy of 73.3% (95% CI, 66.5–78.4) against virologically confirmed dengue and 80.2% (95% CI, 64.3–89.5) against dengue-related hospitalization [40]. These findings showed that TAK-003 could provide clinically meaningful protection against dengue and, importantly, suggested benefit in both previously exposed and dengue-naïve recipients.

Extended follow-up confirmed that protection persisted over time and remained greater against hospitalization than against symptomatic infection. After approximately three years of surveillance, cumulative vaccine efficacy was 62.0% (95% CI, 56.6–66.7) against virologically confirmed dengue and 83.6% (95% CI, 76.8–88.4) against dengue-related hospitalization [48]. Among participants who were seronegative at baseline, efficacy was 54.3% against virologically confirmed dengue and 77.1% against hospitalization. Corresponding estimates among baseline-seropositive participants were 65.0% and 86.0%, respectively. These results indicated that prior dengue exposure enhanced vaccine performance but was not required for clinically relevant protection.

The 4.5-year analysis further demonstrated sustained benefit, with an overall efficacy of 61.2% against virologically confirmed dengue and 84.1% against dengue-related hospitalization [49]. The greater persistence of protection against hospitalization suggests that the vaccine may have a particularly important role in reducing the most clinically consequential outcomes of dengue. No new major safety concerns emerged during prolonged follow-up, and protection against hospitalization remained evident in both seropositive and seronegative participants.

A particularly relevant finding from the TAK-003 development program was the absence of a clear signal of vaccine-associated disease enhancement. In contrast to the experience with CYD-TDV, long-term analyses did not demonstrate an increased incidence of hospitalized or severe dengue among baseline-seronegative vaccine recipients [14]. This observation has been central to the favorable benefit–risk assessment of TAK-003 and supports its use without mandatory pre-vaccination serological screening in appropriately selected epidemiological settings. Moreover, a recent communication by the vaccine manufacturer has also indicated persistent protection against dengue and dengue-related hospitalization without an emerging signal suggestive of ADE up to seven years from administration [50].

The overall safety profile of TAK-003 has remained favorable across phase I, II, and III studies. Most solicited adverse events were mild or moderate and included injection-site pain, headache, fatigue, malaise, myalgia, and transient fever [51]. Serious adverse events occurred at similar frequencies in vaccine and placebo groups, and long-term follow-up did not show an increased overall risk of severe dengue, hospitalization, or vaccine-enhanced disease among vaccinated participants [48,49]. The favorable efficacy profile of TAK-003, combined with the absence of a requirement for pre-vaccination serological screening and its generally acceptable safety and tolerability, suggests that this vaccine may represent a cost-effective strategy for dengue prevention, particularly in DENV-endemic settings. In a dynamic transmission model based on efficacy data from the DEN-301 trial and evaluated from a societal perspective over a 20-year horizon, routine TAK-003 vaccination was projected to prevent 41–57% of symptomatic dengue cases and 47–70% of dengue-related hospitalizations. The vaccination strategy associated with the greatest public health impact, consisting of routine immunization at 6 years of age combined with 10 catch-up cohorts, was estimated to avert 104,415 disability-adjusted life years (DALYs) and generate approximately US$1.79 billion in societal cost savings. A more pragmatic strategy integrating TAK-003 into the existing HPV vaccination program at 11 years of age was projected to reduce symptomatic dengue by 44% and hospitalizations by 53%, while yielding approximately US$1.35 billion in savings over two decades. Sensitivity analyses demonstrated the robustness of these findings across a wide range of assumptions [52].

Despite these favorable findings, important limitations remain. Serotype-specific analyses have demonstrated heterogeneous efficacy across the four DENV serotypes. Protection has been consistently highest against DENV-2, the backbone serotype, with efficacy estimates exceeding 90% during the early years of follow-up [40,48]. Substantial protection has also been observed against DENV-1. In contrast, estimates for DENV-3 and particularly DENV-4 have been less robust and, in some analyses, imprecise because of the limited number of endpoint cases caused by these serotypes [51].

Longer-term findings have raised uncertainty regarding the magnitude and durability of protection against DENV-3 and DENV-4, particularly among participants who were seronegative before vaccination. Although no consistent signal of increased severe disease has emerged, the available evidence is insufficient to establish equally strong and durable protection against all four serotypes [12,13,37]. These observations underscore the importance of distinguishing overall tetravalent immunogenicity from demonstrated clinical efficacy against each individual serotype.

For this reason, continued post-licensure surveillance is essential. Effectiveness studies should assess vaccine performance according to baseline serostatus, age, time since vaccination, circulating serotype and genotype, and local transmission intensity. Such monitoring is particularly important in regions where DENV-3 or DENV-4 predominates and where pre-existing population immunity may differ from that of the TIDES trial populations [6,14].

Notwithstanding these uncertainties, TAK-003 has obtained regulatory authorization in multiple jurisdictions without a requirement for routine pre-vaccination serological testing. This represents an important programmatic advantage over CYD-TDV and substantially simplifies vaccine implementation. However, the absence of mandatory serological screening does not eliminate the need for careful selection of target populations and epidemiological settings.

The public health relevance of TAK-003 is considerable. On the basis of available efficacy, safety, and immunogenicity data, the World Health Organization recommends consideration of TAK-003 for children aged 6–16 years living in settings with high dengue transmission intensity and a substantial burden of hospitalization and severe disease [6,14]. Regulatory approvals in countries across Asia, Latin America, and Europe have made TAK-003 the most broadly deployable dengue vaccine currently available.

Overall, TAK-003 represents an important advance in dengue prevention because it provides sustained protection against symptomatic disease and, more consistently, against hospitalization in both seropositive and seronegative individuals. Nevertheless, uncertainties regarding serotype-specific effectiveness, particularly against DENV-3 and DENV-4 in dengue-naïve populations, remain clinically relevant. Its optimal use should therefore be guided by local epidemiology, disease burden, circulating serotypes, age-specific risk, and continued pharmacovigilance and effectiveness monitoring.

In July 2026, the Central Drugs Standard Control Organisation (CDSCO), under the Drug Controller General of India (DCGI), granted marketing authorization for Qdenga for the prevention of dengue in individuals aged 4–60 years. This represented the first regulatory approval of a dengue vaccine in India. The authorization permits vaccination irrespective of previous dengue exposure and does not require pre-vaccination serological testing [53].

### 4.3. Butantan-DV

Butantan-DV is a live-attenuated tetravalent dengue vaccine developed by Instituto Butantan in São Paulo, Brazil, in collaboration with the US National Institutes of Health. It is derived from the NIH TV003 platform, which was designed to induce broad immunity against all four DENV serotypes after a single administration. The vaccine contains attenuated DENV-1, DENV-3, and DENV-4 strains together with a chimeric DENV-2 component constructed on an attenuated DENV-4 backbone.

Following inoculation, the vaccine viruses undergo limited replication, providing sustained antigenic stimulation that more closely resembles natural infection than non-replicating vaccine platforms. This transient replication is intended to promote innate immune activation and the development of neutralizing antibodies, memory B cells, and dengue-specific T-cell responses. The single-dose formulation is therefore designed to induce durable tetravalent immunity while maintaining an acceptable safety profile. The immunological and virological basis of this approach was established during the development of the NIH TV003 vaccine and subsequently evaluated in the clinical development program of Butantan-DV [54,55].

Early-phase clinical studies supported the feasibility of this strategy. Phase I investigations showed that a single dose was generally well tolerated and induced neutralizing antibody responses against all four DENV serotypes. Transient detection of vaccine-virus replication was consistent with biologically meaningful antigenic stimulation and the induction of immune memory. Subsequent phase II studies confirmed broad immunogenicity, with approximately 80% of participants developing neutralizing antibodies against all four serotypes after a single vaccination. Responses were observed in both dengue-seronegative and dengue-seropositive individuals, supporting further evaluation of the vaccine without mandatory pre-vaccination serological screening [56].

The immunogenicity demonstrated in early-phase studies translated into substantial clinical protection in a pivotal randomized, double-blind, placebo-controlled phase III trial conducted in Brazil. The study enrolled 16,235 participants aged 2–59 years who received either a single dose of Butantan-DV or a placebo. After two years of follow-up, overall vaccine efficacy against virologically confirmed symptomatic dengue was 79.6% (95% CI, 70.0–86.3%). Efficacy was 73.6% among participants who were seronegative at baseline and 89.2% among those who were seropositive.

Efficacy was broadly consistent across age groups, reaching 80.1% among children aged 2–6 years, 77.8% among participants aged 7–17 years, and 90.0% among adults aged 18–59 years. Serotype-specific efficacy was 89.5% against DENV-1 and 69.6% against DENV-2. However, efficacy against DENV-3 and DENV-4 could not be reliably estimated because these serotypes circulated only minimally during the study period. Vaccine-related serious adverse events were uncommon, occurring in fewer than 0.1% of participants, and no cases of vaccine-associated severe dengue were identified during the initial follow-up [57].

Extended follow-up indicated that protection persisted, although efficacy against symptomatic infection declined over time. At a mean follow-up of 3.7 years, vaccine efficacy against virologically confirmed dengue caused by DENV-1 or DENV-2 was 67.3% [58]. Five-year follow-up subsequently showed an overall efficacy of 65.0% against symptomatic dengue. Efficacy remained higher among participants with previous dengue exposure than among dengue-naïve participants, at 77.1% and 58.9%, respectively.

Protection against more clinically consequential outcomes remained comparatively robust. Efficacy against severe dengue or dengue with warning signs was 80.5%, and no vaccinated participant required hospitalization for dengue during long-term follow-up. Serotype-specific efficacy remained substantial against DENV-1 and DENV-2, at 73.0% and 55.7%, respectively. However, the continued scarcity of DENV-3 and DENV-4 cases prevented reliable assessment of protection against these serotypes [59].

These findings suggest that Butantan-DV may represent an important advance in dengue prevention. Its principal potential advantages include a single-dose schedule, clinically meaningful efficacy in both seropositive and seronegative individuals, and sustained protection against severe disease. Nevertheless, the available efficacy evidence is derived predominantly from circulation of DENV-1 and DENV-2. Protection against DENV-3 and DENV-4, particularly in dengue-naïve populations, therefore remains insufficiently characterized.

On the basis of the phase III findings, the Brazilian regulatory authority ANVISA approved Butantan-DV in 2025 for the prevention of dengue in individuals aged 12–59 years. Following its introduction into routine use and the administration of more than 500,000 doses during the initial rollout in 2026, pharmacovigilance systems identified a small number of serious and unexpected clinical events that had not been observed during prelicensure trials.

Reports from ANVISA and the Brazilian Ministry of Health described 42 cases involving manifestations such as severe abdominal pain, persistent vomiting, and bleeding, some of which resembled clinical features of dengue. Three events were classified as severe, and two deaths were reported among vaccine recipients. However, the occurrence of an event after vaccination does not establish causality, and regulatory authorities emphasized that a causal relationship between Butantan-DV and these outcomes had not been demonstrated. In particular, it was highlighted that these events did not represent ADE or vaccine-enhanced dengue. The reports prompted a precautionary interruption of vaccination while detailed clinical, epidemiological, and pharmacovigilance investigations were undertaken [60,61].

These post-marketing observations should be interpreted cautiously. Rare adverse events may not be detected in prelicensure trials because even large phase III studies have limited power to identify events occurring at very low frequencies. Conversely, events temporally associated with vaccination may reflect background disease, coincidental infections, or unrelated medical conditions. Careful comparison of observed and expected event rates, assessment of biological plausibility, review of individual clinical records, and evaluation of potential clustering by age, location, vaccine lot, or time since vaccination are therefore essential before conclusions regarding causality can be drawn.

Butantan-DV has not yet received WHO prequalification or broad international regulatory authorization. Its future role will depend on the outcome of ongoing pharmacovigilance assessments, confirmation of long-term safety, and the accumulation of additional effectiveness data under routine-use conditions. Evidence regarding protection against DENV-3 and DENV-4 will also be necessary to determine whether the vaccine provides consistently balanced protection across different epidemiological settings.

Provided that its favorable efficacy profile is confirmed and current safety uncertainties are satisfactorily resolved, Butantan-DV could substantially facilitate dengue vaccination programs. A single-dose schedule may improve uptake, reduce logistical complexity, lower delivery costs, and limit the loss to follow-up associated with multidose regimens. Nevertheless, broader international recommendations will require independent regulatory assessment, continued active safety surveillance, longer-term effectiveness data, and more complete characterization of serotype-specific protection.

Although direct comparison between trials should be undertaken cautiously because of differences in study populations, baseline serostatus, circulating serotypes, endpoints, and duration of follow-up, Table 3 summarizes the principal efficacy and long-term findings for the three authorized vaccines [28,29,30,31,32,33,34,35,36,37,38,39,40,41,42,43,44,45,46,47,48,49,50,51,52,53,54,55,56,57,58,59].

## 5. Vaccines in Advanced or Early Clinical Development

Although CYD-TDV (Dengvaxia^®^), TAK-003 (Qdenga^®^), and Butantan-DV are the only dengue vaccines to have reached regulatory authorization, several additional vaccine platforms have progressed through preclinical or early clinical development. These approaches seek to improve safety, broaden tetravalent immunity, simplify vaccination schedules, and overcome the limitations of currently available live-attenuated vaccines. However, most remain at an investigational stage, and none has yet generated phase III efficacy data comparable to those available for licensed products.

### 5.1. NIH TV003/TV005

The NIH TV003/TV005 vaccine represents the direct precursor of Butantan-DV and is based on the same live-attenuated tetravalent platform. It contains attenuated DENV-1, DENV-3, and DENV-4 strains together with a chimeric DENV-2 component and was designed to induce balanced immunity after a single administration. TV005 differs from TV003 primarily in the relative dose of the DENV-2 component.

In phase I and phase II studies, a single dose induced neutralizing antibody responses against all four serotypes in most flavivirus-naïve recipients and generated both humoral and cellular immune responses. Controlled human infection studies further demonstrated protection against the experimental DENV-2 challenge, providing important proof of concept for the platform [55,62]. Although TV003/TV005 has not itself progressed to broad regulatory authorization, its development established the scientific foundation for Butantan-DV and remains an important model for single-dose live-attenuated dengue vaccination.

### 5.2. Purified Inactivated Dengue Vaccines

Purified inactivated tetravalent dengue vaccines were initially developed by the Walter Reed Army Institute of Research, GlaxoSmithKline (London, UK), and collaborating institutions. These vaccines contain chemically inactivated whole DENV particles and therefore cannot replicate in vaccine recipients. Their non-replicating nature offers potential safety advantages, particularly for individuals in whom live vaccination may be contraindicated, but also limits the duration and magnitude of antigenic stimulation.

In preclinical studies, formulations adjuvanted with aluminum hydroxide, AS01E, or AS03B induced neutralizing antibodies against all four serotypes in rhesus macaques and reduced viremia after viral challenge. These findings supported progression to human evaluation. However, robust antibody responses generally required potent adjuvants and repeated administration, suggesting that a single dose was unlikely to generate sustained protection.

Phase I randomized trials in healthy flavivirus-naïve adults evaluated two-dose schedules administered one month apart [63,64]. The vaccines were generally well tolerated, with adverse events consisting predominantly of mild injection-site reactions and transient systemic symptoms. Neutralizing antibody responses were detectable after vaccination, but titers declined substantially during follow-up, particularly with less immunogenic formulations. Although booster doses elicited anamnestic responses, immune priming alone did not appear sufficient to maintain antibody concentrations likely to provide durable protection.

Accordingly, the principal limitations of purified inactivated dengue vaccines remain their relatively modest immunogenicity, dependence on adjuvants, and need for multidose and potentially booster schedules. Their favorable theoretical safety profile remains attractive, especially for selected populations, but no candidate has yet demonstrated clinical efficacy or advanced to phase III evaluation.

### 5.3. DNA Vaccines

DNA vaccines represent another non-replicating approach to dengue prevention. Most candidates consist of plasmids encoding the premembrane and envelope proteins of one or more DENV serotypes. Following intramuscular administration, host cells express the encoded antigens, thereby promoting endogenous antigen presentation and potentially inducing both antibody and T-cell responses.

In murine and non-human primate models, monovalent and tetravalent DNA constructs generated neutralizing antibodies, elicited dengue-specific cellular responses, and reduced viremia following viral challenge [65]. These findings supported early clinical evaluation of several candidates.

D1ME100, one of the most extensively studied prototypes, is a plasmid DNA vaccine encoding the prM and E proteins of DENV-1. In a phase I dose-escalation study involving healthy flavivirus-naïve adults, participants received 1 mg, 2.5 mg, or 5 mg on days 0, 30, and 90. The vaccine was well tolerated, and no vaccine-related serious adverse events were reported. However, despite administration of three doses, neutralizing antibody responses were modest, measurable in only a minority of participants, and characterized by substantially lower geometric mean titers than those observed after natural infection or live-attenuated vaccination [66]. Dengue-specific cellular responses were detected in some recipients, indicating effective T-cell priming, but the magnitude of the humoral response was insufficient to justify progression to efficacy trials.

Subsequent tetravalent DNA constructs incorporated improved antigen design and broader serotype coverage. Repeated immunization increased antibody concentrations and enhanced T-cell responses, but neutralizing antibody magnitude and durability remained lower than those achieved with live-attenuated platforms such as TV003 or TAK-003 [67]. Heterologous prime–boost strategies combining DNA vaccines with recombinant proteins, viral vectors, or inactivated vaccines have therefore been explored to improve immunogenicity. Although these combinations have produced stronger responses in animal models, they increase programmatic complexity and have not yet generated convincing clinical efficacy data [67].

DNA vaccines offer advantages in manufacturing, stability, safety, and flexibility of antigen design. Nevertheless, their development has been constrained by inadequate induction of durable and balanced tetravalent neutralizing antibodies, the need for repeated dosing, and the absence of evidence of clinical protection.

### 5.4. Viral-Vectored Vaccines

Viral-vectored dengue vaccines use replication-competent or replication-defective vectors, including adenovirus, vaccinia virus, and measles virus, to express selected DENV antigens. Most constructs encode the E and prM proteins, with some also incorporating NS1 or other non-structural antigens. Intracellular expression may enhance major histocompatibility complex class I antigen presentation and promote robust CD8+ T-cell responses.

These platforms offer several theoretical advantages. They avoid direct administration of replication-competent DENV, can be engineered to express antigens from several serotypes, and may induce broader cellular immunity than protein-based vaccines. However, they do not necessarily reproduce the complex antigenic architecture or prolonged multicomponent stimulation associated with natural infection or live-attenuated dengue vaccination.

Preclinical studies have generally shown strong cellular responses and variable neutralizing antibody induction. Human experience remains limited to a small number of early-phase studies, which have reported acceptable tolerability, with predominantly mild local reactions, transient fever, headache, and fatigue. No major platform-specific safety signal has been identified.

The main challenge remains the induction of potent, balanced, and durable neutralizing antibodies against all four serotypes. Viral vectors often generate substantial cellular immunity but comparatively weaker or uneven humoral responses. Pre-existing immunity to the vector may also reduce vaccine take and further complicate repeated administration. Heterologous prime–boost combinations with DNA vaccines, recombinant proteins, or inactivated DENV vaccines have improved immunogenicity in experimental models but increase manufacturing and delivery complexity. To date, no viral-vectored dengue vaccine has demonstrated sufficient clinical efficacy to support progression to phase III evaluation [68].

### 5.5. Virus-like Particle Vaccines

Virus-like particle vaccines have attracted increasing interest because they combine structural mimicry of the virion with the safety of a non-replicating platform. VLPs are formed by the self-assembly of dengue structural proteins into particles that resemble the native viral surface but contain no viral genome and are therefore incapable of replication.

In animal models, monovalent and tetravalent VLP formulations have induced neutralizing antibodies against multiple DENV serotypes and have demonstrated favorable safety profiles. Their capacity to preserve conformational and quaternary epitopes may offer an immunological advantage over isolated recombinant proteins. VLPs also provide potential benefits in manufacturing consistency and eliminate concerns related to vaccine-virus replication or reversion.

Nevertheless, several challenges remain, including achieving balanced antigen expression across all four serotypes, preserving native epitope conformation during manufacturing, and sustaining antibody responses without repeated dosing or potent adjuvants. Human clinical experience remains limited, and no VLP-based dengue vaccine has yet progressed to phase III evaluation [69].

### 5.6. mRNA Vaccines

The rapid development and large-scale deployment of mRNA vaccines during the COVID-19 pandemic have stimulated interest in applying this technology to dengue. Current approaches generally use lipid nanoparticle-formulated mRNA encoding DENV envelope proteins, prM-E constructs, or selected immunogenic epitopes from one or more serotypes.

Preclinical studies in mice and non-human primates have shown that mRNA vaccines can induce neutralizing antibodies and cellular immune responses. The platform offers several potential advantages, including rapid manufacturing, ease of sequence modification, scalable production, and the ability to adjust the relative expression of individual serotype antigens. These features may be especially useful for optimizing tetravalent formulations and responding to emerging information on antigenic diversity [70].

However, dengue presents challenges not encountered with single-antigen viral vaccines. A successful mRNA formulation must generate balanced immunity to all four serotypes while minimizing cross-reactive but weakly neutralizing antibodies. The optimal antigen design, dose, dosing schedule, and need for adjuvant-like innate stimulation remain uncertain. As of 2026, no mRNA-based dengue vaccine has entered late-stage clinical testing, and no clinical efficacy data are available.

### 5.7. Pan-Flavivirus and Universal Vaccine Approaches

A further area of investigation is the development of broadly protective vaccines targeting conserved epitopes shared across dengue and other medically important flaviviruses, including Zika, Japanese encephalitis, West Nile, and yellow fever viruses. These strategies aim to generate cross-reactive immunity through conserved envelope structures, quaternary epitopes, non-structural proteins, or broadly reactive T-cell responses.

Such approaches are conceptually attractive because they could provide protection against several related pathogens and reduce the need for virus-specific vaccination programs. They may also improve preparedness against newly emerging flaviviruses. However, cross-reactivity within the flavivirus family presents substantial immunological challenges. Antibodies that recognize conserved epitopes may not be strongly neutralizing and could theoretically contribute to enhancement, while pre-existing flavivirus immunity may alter subsequent vaccine responses.

Most pan-flavivirus vaccine candidates remain confined to discovery or preclinical development. Considerable work is needed to identify protective antigens, define reliable correlates of broad immunity, demonstrate the absence of enhancement, and establish acceptable regulatory pathways for vaccines intended to protect against multiple pathogens [64].

Collectively, these investigational approaches broaden the technological options for dengue vaccination beyond currently licensed live-attenuated vaccines. Their principal characteristics, stage of development, and remaining scientific and clinical challenges are summarized in Table 4 [55,62,63,64,65,66,67,68,69,70,71].

## 6. Conclusions

The dengue vaccine landscape has evolved substantially, with three live-attenuated vaccines reaching regulatory authorization and a diverse pipeline of alternative technologies under investigation. The available experience demonstrates both the feasibility of dengue vaccination and the continuing importance of baseline serostatus, serotype-specific protection, durability of immunity, and local epidemiology in determining vaccine performance and public health value.

Despite substantial progress, no currently available vaccine fully overcomes the fundamental immunological and virological challenges posed by dengue virus infection. CYD-TDV (Dengvaxia^®^) demonstrated the critical influence of baseline serostatus on vaccine efficacy and safety, with an increased risk of hospitalization and severe dengue in some seronegative recipients. TAK-003 (Qdenga^®^) has improved the benefit–risk profile and can be administered irrespective of previous dengue exposure; however, protection remains heterogeneous across serotypes, and uncertainties persist regarding the durability and balance of protection against all four DENV serotypes. Butantan-DV offers a promising single-dose strategy with demonstrated efficacy in both seropositive and seronegative individuals, but its broader role will depend on confirmation of long-term safety and effectiveness and more complete evidence for DENV-3 and DENV-4. More broadly, all dengue vaccines must contend with antibody-dependent enhancement, serotype-specific immune responses, changes in circulating serotypes, and the absence of clearly defined correlates of protection. Consequently, no current vaccine provides universally durable, balanced, and serostatus-independent protection across all populations and epidemiological settings.

Among currently available vaccines, TAK-003 has the broadest potential for programmatic use because it does not require mandatory pre-vaccination serological testing and has demonstrated sustained protection against symptomatic dengue and, more consistently, dengue-related hospitalization. CYD-TDV remains restricted to individuals with documented previous dengue infection, while Butantan-DV represents a potentially important single-dose alternative requiring further long-term and serotype-specific evaluation.

Population-based dengue vaccination is most readily justified in settings characterized by high and sustained transmission and a substantial burden of hospitalization and severe disease. WHO recommends consideration of TAK-003 for children aged 6–16 years living in areas with high dengue transmission intensity. However, decisions regarding routine vaccination should be based on local epidemiology rather than the presence of competent vectors alone. Relevant factors include sustained DENV circulation, disease incidence and severity, age-specific seroprevalence, healthcare-system burden, vector density, and the capacity to conduct effective surveillance and pharmacovigilance. Vaccination policies should therefore be adapted to national and subnational transmission patterns.

In temperate areas where dengue transmission remains sporadic, universal vaccination is more difficult to justify, and more selective, risk-based strategies are appropriate. Potential target groups include individuals living in areas with recurrent autochthonous transmission and persons with prolonged, repeated, or occupational exposure to highly endemic regions. For travelers, vaccination decisions should consider destination, duration and frequency of travel, individual risk factors, previous dengue exposure, vaccine availability, and regulatory indications. TAK-003 is currently more suitable for travel medicine than CYD-TDV because it can be administered irrespective of prior dengue infection, although its two-dose schedule may limit its usefulness for last-minute travelers. Additional evidence is required regarding effectiveness in seronegative travelers, durability of protection, and optimal candidate selection.

Mediterranean countries warrant particular attention because the widespread establishment of *Aedes albopictus*, increasing temperatures, intense international travel, and recurrent autochthonous transmission may progressively alter regional dengue risk. Nevertheless, local transmission alone does not justify universal vaccination. Decisions should integrate epidemiological, entomological, virological, and economic assessments, together with vaccine price, dosing schedule, delivery costs, screening requirements, health-system capacity, and equity considerations.

Programmatic feasibility is also essential. Primary healthcare services must be able to support vaccine procurement, storage, distribution, trained personnel, follow-up, completion of multidose schedules, adverse-event monitoring, and equitable access. Cold-chain requirements may represent a particular challenge in tropical, remote, and resource-constrained settings, where limitations in refrigeration, electricity, monitoring, and transport may increase costs and contribute to vaccine wastage. Single-dose strategies may offer operational advantages by reducing follow-up requirements.

Continuous surveillance will be essential to guide vaccination policy. Monitoring should include imported and autochthonous cases, hospitalization and severe-disease rates, circulating serotypes and genotypes, age-specific seroprevalence, vector distribution, and evidence of sustained local transmission. These data should be integrated with vaccine effectiveness, safety, feasibility, and cost-effectiveness analyses to identify populations and settings in which vaccination is likely to provide the greatest benefit.

Important knowledge gaps remain, particularly regarding long-term protection, effectiveness against DENV-3 and DENV-4, performance in dengue-naïve populations, booster strategies, correlates of protection, and vaccine safety and effectiveness in older adults, immunocompromised individuals, travelers, and populations in low-transmission settings. Robust post-licensure surveillance and comparative effectiveness studies will therefore remain critical.

Dengue vaccination should not replace vector control, case surveillance, clinical preparedness, or public education but should form part of an integrated prevention strategy adapted to local epidemiology and health-system capacity. The ultimate goal remains the development and rational deployment of vaccines that provide durable and balanced protection against all four serotypes, are safe regardless of previous dengue exposure, and can be implemented effectively in both endemic and emerging-risk settings.

## Figures and Tables

**Table 1:** Principal virological and immunological challenges in dengue vaccine development.

Challenge	Biological Basis	Implications for Vaccine Development
Four antigenically distinct serotypes	DENV comprises DENV-1, DENV-2, DENV-3, and DENV-4. Infection with one serotype generally induces long-term homologous immunity but only transient and incomplete heterologous protection.	Vaccines must induce balanced and durable protection against all four serotypes rather than strong immunity against only one or two.
Antibody-dependent enhancement	Cross-reactive antibodies present at non-neutralizing or sub-neutralizing concentrations may facilitate viral entry into Fcγ receptor-bearing cells and increase the risk of severe dengue	Vaccines must elicit potent, durable, and functionally neutralizing antibodies while minimizing potentially enhancing responses.
Unequal tetravalent immunogenicity	Vaccine components may differ in replication fitness, antigen expression, and immunodominance.	Unbalanced responses may result in weaker protection against specific serotypes and leave recipients susceptible to heterologous infection.
Complex antigenic structure	Potent neutralizing antibodies frequently recognize conformational or quaternary epitopes displayed on mature, intact virions	Vaccine platforms should preserve native antigenic architecture and relevant virion conformations.
Absence of a definitive correlate of protection	Neutralizing antibody titers vary according to assay methods and do not consistently predict clinical protection across serotypes and populations.	Candidate selection and comparison between trials remain difficult; antibody quality and cellular immunity must also be considered.
Role of cellular immunity	CD4+ and CD8+ T cells recognize epitopes in structural and non-structural proteins, including NS1, NS3, and NS5, and contribute to viral clearance and disease modulation.	Platforms inducing both neutralizing antibodies and broad dengue-specific T-cell responses may provide more durable and heterotypic protection.
Baseline dengue serostatus	Prior dengue infection substantially influences vaccine immunogenicity, efficacy, and, for some vaccines, safety.	Vaccine performance must be evaluated separately in seropositive and seronegative individuals.
Viral genetic diversity	Each serotype comprises multiple genotypes and lineages that vary geographically and evolve over time.	Molecular surveillance is required to assess whether circulating strains influence vaccine effectiveness.
Population and epidemiological heterogeneity	Age, transmission intensity, prior flavivirus exposure, circulating serotypes, and host factors differ across settings.	Vaccination strategies cannot be uniformly applied and should be adapted to local epidemiology and target populations.
Programmatic feasibility	Multidose schedules, serological screening, cost, cold-chain requirements, and follow-up may limit uptake.	An ideal vaccine should be safe, affordable, effective after few doses, and suitable for large-scale implementation.

Abbreviations: DENV, dengue virus.

**Table 2:** Main characteristics of licensed or authorized dengue vaccines.

Characteristic	CYD-TDV (Dengvaxia^®^)	TAK-003 (Qdenga^®^)	Butantan-DV
Developer	Sanofi Pasteur	Takeda	Instituto Butantan and US NIH
Vaccine platform	Live-attenuated chimeric tetravalent vaccine using the yellow fever 17D backbone	Live-attenuated tetravalent vaccine based on an attenuated DENV-2 backbone	Live-attenuated tetravalent vaccine derived from the NIH TV003 platform
Vaccine composition	DENV-1–4 prM/E proteins expressed on a yellow fever 17D backbone	Attenuated DENV-2 plus chimeric DENV-1, DENV-3, and DENV-4 components on the DENV-2 backbone	Attenuated DENV-1, DENV-3, and DENV-4 strains plus a chimeric DENV-2 component
Dengue non-structural proteins	Absent; non-structural proteins are derived from yellow fever virus	Present and exclusively derived from DENV-2	Present and originated from DENV-1, DENV-3 and DENV-4
Schedule	Three doses at 0, 6, and 12 months	Two doses three months apart	Single dose
Evidence in seropositive individuals	Strong protection against symptomatic, hospitalized, and severe dengue	Clinically meaningful protection against symptomatic dengue and hospitalization	High efficacy against symptomatic dengue and sustained protection against severe outcomes
Evidence in seronegative individuals	Increased long-term risk of hospitalized and severe dengue; use contraindicated without evidence of previous infection	Protection demonstrated, although efficacy is lower than in seropositive recipients and varies by serotype	Protection demonstrated, but lower than in seropositive individuals; evidence against DENV-3 and DENV-4 remains limited
Pre-vaccination serological screening	Required	Not routinely required	Not routinely required under the Brazilian authorization
Principal efficacy strengths	Effective in previously infected individuals; reduces hospitalization and severe dengue	Sustained protection against hospitalization in seropositive and seronegative recipients	Single-dose administration and efficacy in both seropositive and seronegative recipients
Principal limitations	Risk in seronegative individuals; three-dose schedule; lower efficacy against DENV-2; limited dengue-specific T-cell immunity	Heterogeneous serotype-specific efficacy, with uncertainty regarding DENV-3 and DENV-4 in seronegative populations	Limited data for DENV-3 and DENV-4; restricted international authorization; need for additional post-marketing safety data
Regulatory/public health position	Restricted to individuals with laboratory-confirmed previous dengue infection	Authorized in multiple jurisdictions; considered by WHO for children aged 6–16 years in high-transmission settings	Authorized in Brazil for selected age groups; broader use depends on further regulatory and safety assessment
Key references	[25,26,27,28,29,30,31,32,33,34,35,36]	[40,41,42,43,44,45,46,47,48,49,50,51]	[53,54,55,56,57,58,59,60]

**Table 3:** Principal clinical efficacy and long-term findings for licensed or authorized dengue vaccines.

Vaccine and Study	Population and Schedule	Follow-Up	Principal Efficacy Findings	Key Safety or Interpretative Findings
CYD-TDV, CYD14 [26]	10,275 children aged 2–14 years in five Asian countries; three doses at 0, 6, and 12 months	Primary phase III analysis	Overall efficacy of 56.5% against virologically confirmed symptomatic dengue	Lower efficacy against DENV-2; subsequent follow-up identified increased hospitalization risk in younger recipients
CYD-TDV, CYD15 [29]	20,869 participants aged 9–16 years in Latin America; three-dose schedule	Primary phase III analysis	Overall efficacy of 60.8%	Efficacy was higher in participants with previous dengue exposure
CYD-TDV, pooled and long-term analyses [30,31,32]	Participants from CYD14 and CYD15	Multiyear follow-up	Overall pooled efficacy of 60.3%, increasing to 65.6% in participants aged ≥9 years	Baseline-seronegative recipients had an increased long-term risk of hospitalized and severe dengue; use was subsequently restricted
TAK-003, TIDES primary analysis [40]	20,099 children and adolescents aged 4–16 years in eight endemic countries; two doses three months apart	12 months after the second dose	Efficacy of 73.3% against virologically confirmed dengue and 80.2% against hospitalization	Protection was observed in both seropositive and seronegative participants. Effectiveness against DENV-3 and DENV-4 remains undetermined
TAK-003, three-year analysis [48]	TIDES population	Approximately three years	Efficacy of 62.0% against virologically confirmed dengue and 83.6% against hospitalization	Among seronegative participants, efficacy was 54.3% against dengue and 77.1% against hospitalization. Effectiveness against DENV-3 and DENV-4 re-mains undetermined
TAK-003, 4.5-year analysis [49]	TIDES population	4.5 years	Efficacy of 61.2% against virologically confirmed dengue and 84.1% against hospitalization	Protection against hospitalization remained more durable than protection against symptomatic infection. Effectiveness against DENV-3 and DENV-4 re-mains undetermined
TAK-003, extended follow-up [14,50]	TIDES population	Up to seven years	Persistent protection against dengue and dengue-related hospitalization	No signal suggestive of vaccine-associated disease enhancement was identified. Effectiveness against DENV-3 and DENV-4 re-mains undetermined
Butantan-DV, phase III primary analysis [57]	16,235 participants aged 2–59 years in Brazil; single dose	Two years	Overall efficacy of 79.6%; 73.6% in seronegative and 89.2% in seropositive participants	Efficacy was demonstrated against DENV-1 and DENV-2; insufficient cases were available for DENV-3 and DENV-4
Butantan-DV, extended analysis [58]	Brazilian phase III population	Mean 3.7 years	Efficacy of 67.3% against virologically confirmed DENV-1 or DENV-2 disease	Protection persisted, although efficacy against symptomatic disease declined over time
Butantan-DV, five-year analysis [59]	Brazilian phase III population	Five years	Overall efficacy of 65.0%; 80.5% against severe dengue or dengue with warning signs	No vaccinated participant required hospitalization for dengue; data remained limited for DENV-3 and DENV-4

Abbreviations: DENV, dengue virus; TIDES, Tetravalent Immunization against Dengue Efficacy Study.

**Table 4:** Investigational dengue vaccine platforms and principal development challenges.

Platform or Candidate	Vaccine Strategy	Stage of Development	Main Advantages	Principal Limitations	References
NIH TV003/TV005	Single-dose live-attenuated tetravalent vaccine containing attenuated DENV-1, DENV-3, and DENV-4 and a chimeric DENV-2 component	Phase I/II and controlled human infection studies; precursor of Butantan-DV	Broad tetravalent immunity after one dose; induction of humoral and cellular responses; protection in a DENV-2 challenge model	Limited direct phase III and regulatory development as an independent product	[55,62]
Purified inactivated dengue vaccines	Chemically inactivated whole-virus tetravalent formulations, generally combined with adjuvants	Phase I	Non-replicating platform; favorable theoretical safety profile; potential use when live vaccines are contraindicated	Modest durability of antibody responses; requirement for potent adjuvants, multiple doses, and possibly boosters	[63,64]
DNA vaccines	Plasmids encoding DENV prM/E proteins from one or more serotypes	Preclinical and phase I	Manufacturing simplicity, stability, absence of vaccine-virus replication, and induction of cellular immunity	Weak or inconsistent neutralizing antibody responses; repeated dosing required; no demonstrated clinical efficacy	[65,67]
Viral-vectored vaccines	Adenovirus-, vaccinia-, or measles-virus vectors expressing DENV structural or non-structural antigens	Preclinical and limited early-phase clinical studies	Strong intracellular antigen expression and potential induction of CD8+ T-cell responses	Uneven humoral responses, possible pre-existing vector immunity, and increased complexity of prime-boost strategies	[68]
Virus-like particle vaccines	Self-assembled structural proteins mimicking native virions without viral genetic material	Predominantly preclinical; limited human data	Non-replicating platform; preservation of conformational epitopes; manufacturing consistency	Difficulty achieving balanced tetravalent expression and durable immunity; possible need for adjuvants or repeated dosing	[69]
mRNA vaccines	Lipid nanoparticle-formulated mRNA encoding DENV envelope, prM/E, or selected antigens	Preclinical and early development	Rapid and scalable manufacturing; flexible antigen design; capacity to adjust serotype-specific antigen expression	No clinical efficacy data; optimal antigen design, dose, durability, and balance of tetravalent responses remain uncertain	[70]
Pan-flavivirus vaccines	Conserved envelope epitopes, non-structural proteins, or broadly reactive T-cell targets shared across flaviviruses	Discovery and preclinical development	Potential protection against dengue and other flaviviruses; utility for emerging pathogen preparedness	Risk of poorly neutralizing cross-reactive responses, uncertain correlates of protection, and complex regulatory pathway	[71]

Abbreviations: DENV, dengue virus; NIH, National Institutes of Health; prM, premembrane protein; E, envelope protein.

## Data Availability

No new data were created or analyzed in this study. Data sharing is not applicable to this article.
